# The Effects of Autophagy-Related Genes and lncRNAs in Therapy and Prognosis of Colorectal Cancer

**DOI:** 10.3389/fonc.2021.582040

**Published:** 2021-03-11

**Authors:** Yang Yang, Mingyang Feng, LiangLiang Bai, Mengxi Zhang, Kexun Zhou, Weiting Liao, Wanting Lei, Nan Zhang, Jiaxing Huang, Qiu Li

**Affiliations:** ^1^ Department of Medical Oncology, Cancer Center, West China Hospital, Sichuan University, Sichuan, China; ^2^ West China Biomedical Big Data Center, Sichuan University, Sichuan, China

**Keywords:** colorectal cancer, autophagy, gene, lncRNA, prognosis, immune

## Abstract

Cellular autophagy plays an important role in the occurrence and development of colorectal cancer (CRC). Whether autophagy-related genes and lncRNAs can be used as ideal markers in CRC is still controversial. The purpose of this study is to identify novel treatment and prognosis markers of CRC. We downloaded transcription and clinical data of CRC from the GEO (GSE40967, GSE12954, GSE17536) and TCGA database, screened for differentially autophagy-related genes (DEAGs) and lncRNAs, constructed prognostic model, and analyzed its relationship with immune infiltration. TCGA and GEO datasets (GSE12954 and GSE17536) were used to validate the effect of the model. Oncomine database and Human Protein Atlas verified the expression of DEAGs. We obtained a total of 151 DEAGs in three verification sets collaboratively. Then we constructed a risk prognostic model through Lasso regression to obtain 15 prognostic DEAGs from the training set and verified the risk prognostic model in three verification sets. The low-risk group survived longer than the high-risk group. Age, gender, pathological stage, and TNM stage were related to the prognostic risk of CRC. On the other hand, BRAF status, RFS event, and tumor location are considered as most significant risk factors of CRC in the training set. Furthermore, we found that the immune score of the low-risk group was higher. The content of CD8 + T cells, active NK cells, macrophages M0, macrophages M1, and active dendritic cells was noted more in the high-risk group. The content of plasma cells, resting memory CD4 + T cells, resting NK cells, resting mast cells, and neutrophil cells was higher in the low-risk group. After all, the Oncomine database and immunohistochemistry verified that the expression level of most key autophagy-related genes was consistent with the results that we found. In addition, we obtained six lncRNAs co-expressed with DEAGs from the training set and found that the survival time was longer in the low-risk group. This finding was verified in the verification set and showed same trend to the results mentioned above. In the final analysis, these results indicate that autophagy-related genes and lncRNAs can be used as prognostic and therapeutic markers for CRC.

## Introduction

Colorectal cancer (CRC) is one of the most common malignant tumors of the digestive system, with the top five morbidity and mortality rates in the world (1). At present, the main treatment methods of CRC are surgery, radiotherapy, and chemotherapy. As more research data being gathered, immunotherapy is also gradually applied to treatment of CRC (2). Although the current treatment solutions have extended the survival time of patients with CRC, the prognosis of patients is still not ideal. Nowadays, there are extensive researches regarding to the topics of accurate diagnosis, CRC treatment, and prognostic evaluation tools for the efficacy of tumor molecular drugs about chemotherapy, targeted therapy and immunotherapy in cancer (3). Cell autophagy exists in human body cells as an important biological process. The genetic information of cell autophagy can participate in the development of CRC and has good application prospects in diagnosis and treatment (4). However, whether cell autophagy has the potential for therapeutic efficacy and prognosis evaluation of CRC is not yet known.

Cellular autophagy can degrade its own structure through lysosomal phagocytosis, which is divided into large autophagy, small autophagy, and molecular chaperone-mediated autophagy which has a wide range of biological effects. In the early stage of tumor growth, autophagy plays a role in suppressing cancer (5). As the tumor grows, in order to adapt to nutritional deficiencies and hypoxic conditions, autophagy starts to come back to the tumor cells. At this stage, autophagy plays a role in protecting the tumor (6). Meanwhile, there are many important genes regulating these processes. PINK1 was also identified as a tumor suppressor gene, indicating that mitochondrial autophagy has a certain role in promoting cancer (7). The relative expression levels of Beclin-1 and Atg7, which are the key proteins associated with the initial formation of autophagosomes in the tumor tissues of patients (8). Also, ATG4B can cleave microtubule-associated protein light chain 3 and other ATG8 adaptor proteins, which are necessary steps for the subsequent lipidation, autophagosome binding, and maturation of autophagy. At the same time, ATG4B inhibitors can block autophagy and promote the death of tumor cells (9).

In view of the dual role of autophagy as a tumor suppressor and tumor promoter, upregulation of autophagy and inhibition of autophagy can be used as potential therapeutic and prognostic strategies in different types of tumors (10). Similarly, autophagy may exhibit dual effects of inhibition and promotion at different stages of CRC development, and the relationship between autophagy and CRC treatment and prognosis is also very close. Studies have shown that the mutation, reduction, or deletion of Beclin1 will reduce the autophagy of cells and promote the occurrence and development of CRC (4). Both UVRAG and Ambra1 proteins can be combined with Beclin1 to induce autophagy in microsatellite unstable CRC. Frameshift mutation UVRAG can counteract Beclin1 induced autophagy and DNA repair and other tumor suppressing functions (11). Prox1 not only promotes the survival and metastasis of CRC cells by inducing autophagy and inhibiting apoptosis, but also promotes the survival of tumor cells in hypoxic regions by increasing autophagy (12). Christensen et al. (13) found that patients with CRC have a high mutation rate of the KRAS (Kirsten rat sarcoma viral oncogene homolog) gene. KRAS mutations promote autophagy through the ERK signaling pathway, which helps CRC cells survive under starvation conditions Drug treatment also affected the autophagy activity of CRC cells. Also, Fluorouracil (5-FU) induces increased autophagy in colon cancer cells with p53 deletion or mutation leading to increased drug resistance (14). Some scholars have studied the effect of autophagy on the efficacy of oxaliplatin and found that L-OHP can induce autophagy in CRC cells to protect CRC cells from apoptosis (15). Other studies have shown that certain drugs can exert anti-CRC effects by inducing autophagy. For instance, Salvianolic acid B is a new type of autophagy inducer that can induce autophagy by inhibiting the AKT/mTOR pathway and inhibit CRC growth (16). On the other hand, structural modification of berberine can increase its antitumor effect on CRC cells, and this new berberine derivative can trigger non-apoptotic death of CRC cells by inducing autophagy (17). Therefore, the role of autophagy in CRC is still controversial.

Although there are studies that showing the effectiveness of cell autophagy and its relationship to CRC, the role of autophagy-related genes and lncRNA in the immune and prognosis of CRC is not completely clear. The analysis method of bioinformatics can comprehensively analyze the whole genome data of all samples in the public database to obtain more objective results (18). Therefore, we download the transcriptome and clinical information of CRC patient samples from GEO and TCGA databases to construct CRC cancer risk prognosis model based on differentially expressed autophagy-related genes (DEAGs) and lncRNAs. Our findings can be used to evaluate the efficacy of patients with chemotherapy and immunotherapy. Also, it can screened out potential markers that might be used as diagnosis and treatment tool of CRC to provide a basis for the clinical application of autophagy-related molecules in CRC.

## Materials and Methods

### Data Collection and Collation

We download transcriptome data and clinical data of CRC from GEO database (GSE40967, GSE12954, GSE17536) and TCGA database (https://portal.gdc.cancer.gov/). GSE40967 from GEO datasets were selected by search term: colorectal cancer, survival, and following the criteria:(1) The samples were only from *Homo sapiens*; (2) The data set must contain expression profile of array and clinical survival data; (3) Raw data could be obtained; (4) The data set must contain tumor and/or normal samples. GSE40967 data including 566 tumor samples and 19 normal samples is used as the training set; TCGA data (including 488 tumor samples and 52 normal samples), GSE12954 (62 tumor samples), and GSE17536 (177 tumor samples) were used as the verification set. Then all the gene id was transformed. According to the name of 222 autophagy genes (AGs) in the autophagy gene database (http://www.autophagy.lu/clustering/index.html), the AGs in the transcriptome were obtained and analyzed for differential expression by R package “edgeR” with the significance threshold “p < 0.05” and “logFC fold change>2.” At the same time, biotype screening of lncRNA was also used for differential analysis by R package “edgeR” with the significance threshold “p < 0.05,” and the Weighted Gene Co-expression Network Analysis was used to analyze and screen out differentially autophagy-related lncRNAs (DAR-lncRNAs) by R package “WGCNA”. Cytoscape draws a co-expression network diagram of AGs and lncRNA.

### Gene Enrichment Analysis

We used WebGestalt (http://www.webgestalt.org/) to analyze enrichment level of all DEAGs in biological process, cellular component, molecular function, and signaling pathway of CRC. Gene Set Enrichment Analysis (GSEA) was performed enrichment analysis on all AGs about risk prognosis model signaling pathways of CRC. All enrichment results were selected with the significance threshold “p < 0.05.”

### Immune Cell Infiltration Calculation and Tumor Microenvironment Score

We calculated the scores of 22 immune cells by CIRBESORT to evaluate the infiltration of immune cells by R package “e1071,” “parallel,” and “preprocessCore.” Then we calculated the matrix score and immune score of the tumor microenvironment to evaluate the content of immune components in the tumor microenvironment.

### Cluster Analysis

Through unsupervised clustering by R package “Consensus ClusterPlus,” we clustered CRC samples based on the similarity of DEAGs to evaluate the optimal number of clusters according to the maximum area under the CDF curve (http://www.bioconductor.org/). Principal component analysis (PCA) analyzed the expression patterns of DEAGs in each cluster. Then we compared the survival time and the changes of clinical factors of each cluster (age, gender, pathological stage, TNM stage, whether there are progression events, gene mutations, and mismatch repair) according to the clusters, and present the results with heat maps (R package “pheatmap”). Additionally, the immune cells infiltration degree and the tumor microenvironment score of the two groups were compared.

### Construction of Risk Prognosis Model With Differentially Autophagy-Related Genes

We screened the DEAGs related to prognosis by unicox with the significance threshold “p < 0.05” from GEO data, and then built risk prognosis model by Lasso regression to reduce the dimension (The calculation formula of the risk prognosis model = the expression level of gene 1*genecoef 1 + the expression level of gene 2*genecoef 2 +…+ the expression level of gene N + genecoef N), and divided the tumor samples into high-risk group and low-risk group according to the median of the risk score (19). Then the difference of the survival and clinical factors of the two groups (age, gender, pathological stage, TNM stage, whether there are progression-stage survival events, gene mutations, mismatch repair) in the two groups was compared by Chi-squared test with the significance threshold “p < 0.05,” and present the results with a heat map. Univariate cox and multivariate cox determined the correlation between clinical factors and prognostic risk and were visualized by forest plot by R package “survival” and “forestplot.” The nomogram baseline was also used to present the multivariate regression results. The relationship between model sensitivity and specificity was evaluated by area under the curve (AUC) of ROC curve. When AUC was greater than 0.5, the model had better specificity and sensitivity. Additionally, the immune cells infiltration degree and the tumor microenvironment score of the two groups were compared with the significance threshold “p <0.05.”

### Validation of Model and Gene Expression With Differentially Autophagy-Related Genes

We used expression and clinical data of CRC from TCGA database to verify the effectiveness of the model. The expression of DEAGs in TCGA database was substituted into the calculation formula of the risk prognosis model. The AGs obtained from the TCGA data were intersected with the data from the GEO training set. The model was also constructed using unicox and Lasso regression to evaluate whether the results were consistent and compared the difference of overall survival. The expression of DEARs related to prognosis was verified in the Oncomine database (https://www.oncomine.org/resource/main.html) and Human Protein Atlas database (https://www.proteinatlas.org/).

### Construction and Validation of Risk Prognosis Model With Differentially Autophagy-Related lncRNA

Similarly, we screened DAR-lncRNA related to the prognosis by unicox with the significance threshold “p < 0.05” and performed Lasso regression analysis from the training set to obtain a risk score. Based on the median risk score, the samples were also divided into high-risk group and low-risk group, and the overall survival of the two groups was compared. The ROC curve measured the sensitivity and specificity of the model. The validation set verified the effect of the model.

### Statistical Analysis

All data statistics were implemented using R-3.6.3. The statistical significance of all results was measured by p < 0.05. The figures were shown by *P < 0.05, **P < 0.01, ***P < 0.001, and ****P < 0.0001.

## Results

### Analysis Results of Differentially Autophagy-Related Genes in Colorectal Cancer

**Figure 1:**
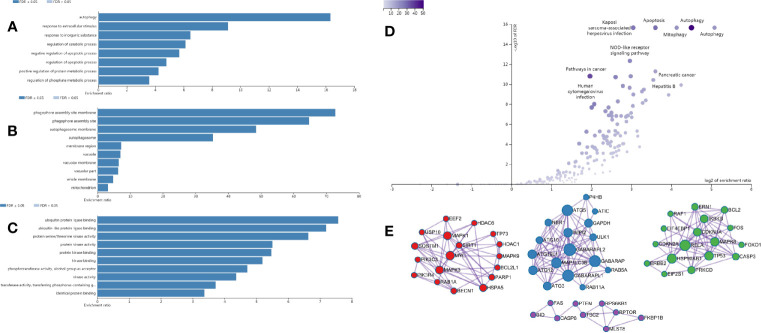
Enrichment analysis results of DEAGs in CRC from the WebGestalt website. **(A)** The biological process of GO analysis in CRC. **(B)** The cellular component of GO analysis in CRC. **(C)** The molecular function of GO analysis in CRC. **(D)** The signaling pathway of KEGG analysis in CRC. **(E)** The protein-protein interaction network of DEAGs in CRC.

We performed differential expression analysis from the transcriptome data downloaded from the GEO database and the TCGA database, and obtained 151 DEAGs from the intersection, including 77 DEAGs with high expression and 74 DEAGs with low expression. All results were visualized by heatmap (**Figure S1A**). Gene enrichment analysis and signaling pathways by WebGestalt showed that DEAGs played an important role in autophagy-related biological processes (**Figures 1A–C**) and signaling pathways (**Figure 1D**) in CRC cells. The protein interaction network analysis was shown in the **Figure 1E**.

### Unsupervised Cluster Analysis and Clinical Characteristics of Differentially Autophagy-Related Genes in Colorectal Cancer

**Figure 2:**
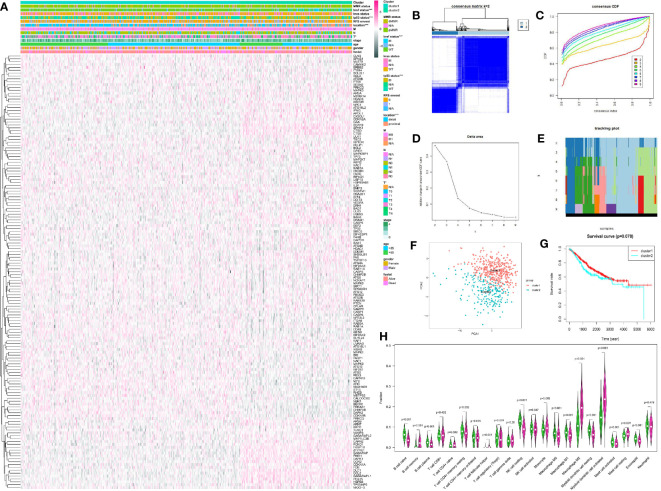
Unsupervised cluster analysis of CRC by R package “ConsensusClusterPlus.” **(A)** The expression of DEAGs in GEO database and clinical characteristics in two clusters by R package “pheatmap.” **(B)** Consensus matrix of unsupervised cluster analysis (k = 2). **(C)** Consensus CDF curve of unsupervised cluster analysis. **(D)** Delta area under CDF curve of unsupervised cluster analysis. **(E)** Tracking plot of unsupervised cluster analysis. **(F)** Principal component analysis of unsupervised cluster analysis. **(G)** Survival curve of two main clusters. **(H)** 22 types of immune cells infiltration in the two clusters. Green showed cluster1 and red showed cluster2. *P < 0.05, **P < 0.01, ***P < 0.001, and ****P < 0.0001.

We classified CRC patients with different AGs quality based on DEAGs in training set. Two different expression patterns were finally determined using unsupervised clustering based on the CDF curve, including 374 cases in cluster1 and 192 cases in cluster1 (**Figures 2B–E**). Principal component analysis showed the results of two clusters (**Figure 2F**). Prognostic analysis of the two main clusters revealed a slightly prominent survival advantage in cluster1, but it was not statistically significant. (**Figure 2G** **).** Comparison of clinical factors in the two clusters showed that about T stage T1-T2 was better in cluster1, and T3-T4 was more prominent in cluster2. In the tumor location, the proximal location occurred more in cluster1, and the distal location occurred more in cluster2. The tp53 gene status and BRAF gene status were particularly prominent in the wild-type of cluster1 and the superiority of mutated-type in cluster2. The clinical factors such as age, gender, and survival status were not significantly different between the two clusters (**Figure 2A**). The patients in the TCGA data were also classified into two clusters. However, the survival of two clusters also had no significant difference (**Figure S2**).

### Immune Score and Immune Cell Infiltration of Each Cluster in Colorectal Cancer

Meanwhile, we compared the immune scores of the two clusters and the infiltration of 22 immune cells. The immune score did not show a significant difference between the two clusters (**Figure S3**). However, there were some significant difference about 22 types of immune cells in the two clusters. The infiltration level of B cell naïve, plasma cells, T cell CD4+ memory resting, Tregs, NK cell resting, NK cell activated, Macrophage M0, Macrophage M1, Myeloid dendritic cell resting, Mast cell activated, and Eosinophil was higher in cluster1. And the infiltration level of B cell memory, T cell follicular helper, Monocyte, Macrophage M2, dendritic cell activated, and Mast cell resting was higher in cluster 2 (**Figure 2H** **).**

### Construction and Verification of Risk Prognosis Model in Colorectal Cancer

**Figure 3:**
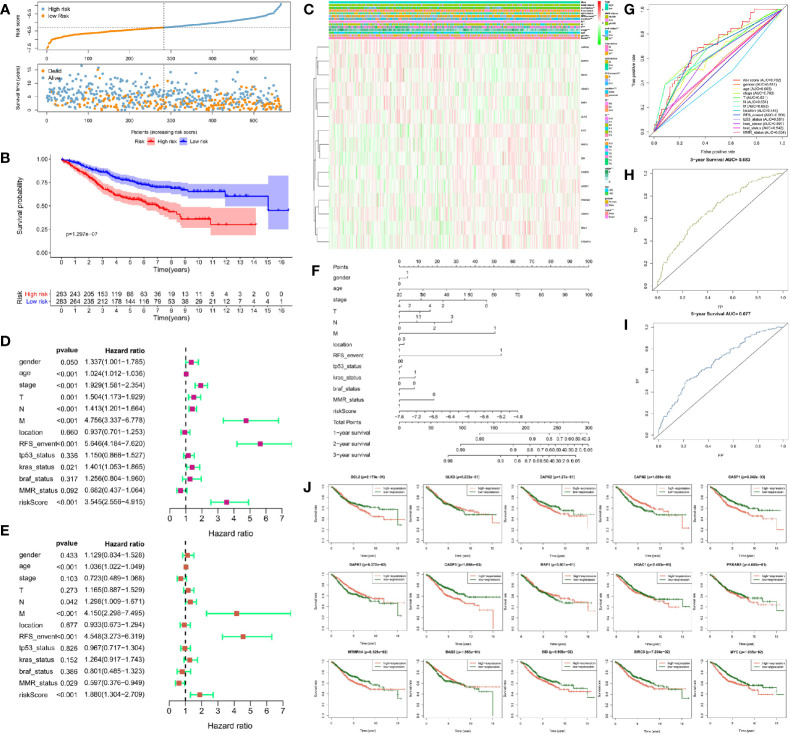
Risk prognosis model construction of 15 prognostic risk-related DEAGs in GEO data by unicox and Lasso regression. **(A)** The distribution of risk score and the scatterplot of the relationship between risk scores and survival time by R package “ggplot.” **(B)** Survival curve comparing high-risk and low-risk groups by R package “survival.” **(C)** Heat map of prognostic DEAGs and clinical parameters at high-risk and low-risk groups by R package “pheatmap.” **(D)** The univariate cox forest map of 13 clinical characteristics in the training set by R package “survival” and “forestplot.” **(E)** The multivariate cox forest plot of 13 clinical characteristics in the training set by R package “survival” and “forestplot.” **(F)** The nomogram baseline of multivariate cox analysis by R package “rms.” **(G)** ROC curve of risk sore and other clinical characteristics by R package “survivalROC.” **(H)** ROC curve of 3-year survival. **(I)** ROC curve of 5-year survival by R package “survivalROC.” **(J)** The survival curve of 15 prognostic risk-related DEAGs expression by R package “survival.” *P < 0.05, **P < 0.01, ***P < 0.001, and ****P < 0.0001.

**Figure 4:**
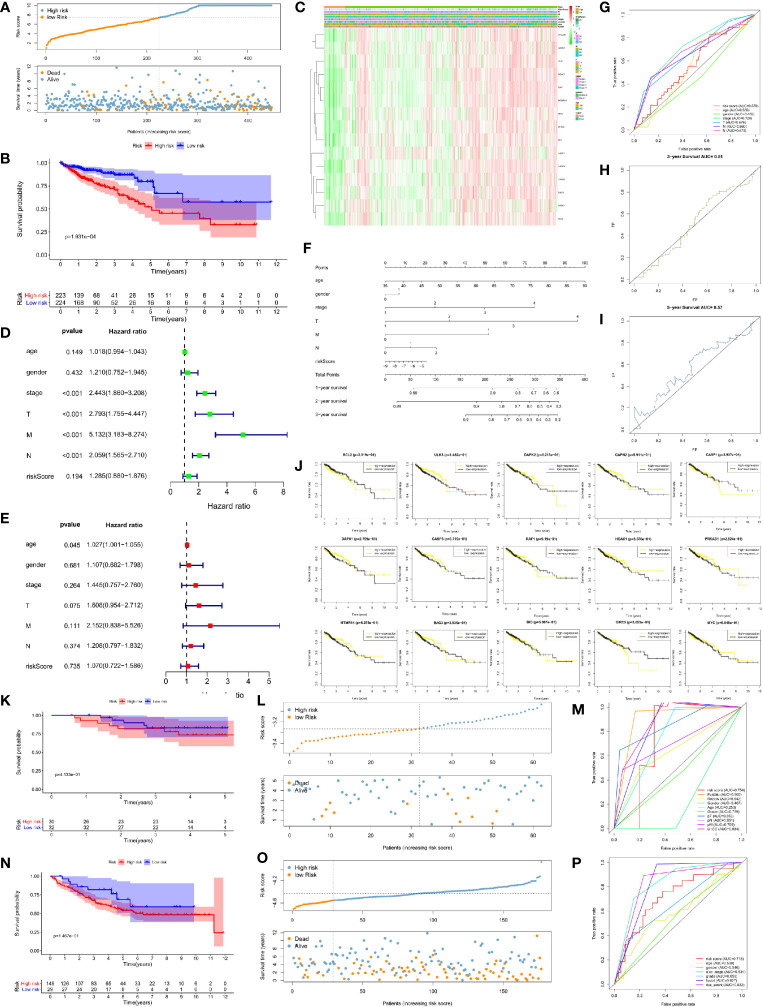
Risk prognosis model verification of 15 prognostic risk-related DEAGs in validation sets with the formula of risk model. **(A)** The distribution of risk score and the scatterplot of the relationship between risk scores and survival time in TCGA data by R package “ggplot.” **(B)** Survival curve comparing high-risk and low-risk groups in TCGA data by R package “survival.” **(C)** Heat map of prognostic DEAGs and clinical parameters at high-risk and low-risk groups in TCGA data by R package “pheatmap.” **(D)** The univariate cox forest map of clinical characteristics in TCGA data by R package “survival” and “forestplot.” **(E)** The multivariate cox forest plot of clinical characteristics in TCGA data by R package “survival” and “forestplot.” **(F)** The nomogram baseline of multivariate cox analysis in TCGA data by R package “rms.” **(G)** ROC curve of risk sore and other clinical characteristics in TCGA data by R package “survivalROC.” **(H)** ROC curve of 3-year survival in TCGA data. **(I)** ROC curve of 5-year survival in TCGA data by R package “survivalROC.” **(J)** The survival curve of 15 prognostic risk-related DEAGs expression in TCGA data by R package “survival.” **(K)** Survival curve comparing high-risk and low-risk groups in GSE12954 set by R package “survival.” **(L)** The distribution of risk score and the scatterplot of the relationship between risk scores and survival time in GSE12954 set by R package “ggplot.” **(M)** ROC curve of risk sore and other clinical characteristics in GSE12954 set by R package “survivalROC.” **(N)** Survival curve comparing high-risk and low-risk groups in GSE17536 set by R package “survival.” **(O)** The distribution of risk score and the scatterplot of the relationship between risk scores and survival time in GSE12954 set by R package “ggplot.” **(P)** ROC curve of risk sore and other clinical characteristics in GSE17536 set by R package “survivalROC.” *P < 0.05, **P < 0.01, ***P < 0.001, and ****P < 0.0001.

We used the unicox to filter prognosis-related DEAGs and then Lasso regression to construct a risk prognosis model. We obtained 15 prognostic risk-related DEAGs (Risk model = DAPK1*0.089824 + CAPN2*0.041346 + RAF1*0.118416 − MYC*−0.13661 − BIRC5*0.13171 − PRKAB1*0.06286 BCL2*0.25849 − CASP3*0.17743 − CASP1*0.05293 + BAG3*0.006582 + ULK3*0.196998 − MTMR14*0.17958 − DAPK2*0.00886 − BID*0.14231 − HDAC1*0.02745), and divided all samples into high risk group and low risk group (**Figure S4**). Consistent with the prediction, the data of the GSE40967 training set (HR = 0.462158, 95% CI = 0.347134–0.615297) (**Figures 3A, B**) and the validation sets including TCGA set (HR = 0.453087, 95% CI = 0.284488–0.721604) (**Figures 4A, B**), GSE12954 set (HR = 0.622264, 95% CI = 0.200154–1.934574) **(** **Figures 4K, L** **)** and GSE17536 set (HR = 0.585198, 95% CI = 0.319431–1.072084) **(** **Figures 4N, O** **)** showed that the survival advantage was both more prominent in the low-risk group. Furthermore, in the low-risk group of the training set, the survival status, pathological stage I-II, T1-2, no lymph node metastasis, no distant metastasis, distant occurrence location, no RFS event and wild-type BRAF occurred more times (**Figure 3C**). However, this significant change was not shown in the verification set (**Figure 4C**).

At the same time, we screened other prognostic risk factors through univariate and multivariate cox analysis. The forest map of univariate cox in the training set indicated gender, age, pathological stage, T stage, N stage, M stage, the occurrence of RFS events, wild-type KRAS,and risk model scores were all significant risk factors for the prognosis of CRC. Tumor location and dMMR were protective factors but not statistically significant (**Figure 3D**). The univariate cox analysis of the validation set only had that pathological stage, T stage, N stage, and M stage were meaningful risk factors, and no other clinical features showed meaningful changes (**Figure 4D**). The multivariate cox forest plot of the training set showed that age, N stage, M stage, RFS event occurrence, and risk model score were also significant risk factors for the prognosis of CRC. Instead of the result in the univariate cox analysis, dMMR was a meaningful protection factor. The remaining factors showed no statistical significance (**Figure 3E**). The multivariate cox analysis of the validation set only showed that age (>65 year-old) was a meaningful risk factor (**Figure 4E**). The nomogram baseline also calculated and presented the relationship between the 1-year, 2-year, and 3-year survival scores and clinical factors, showing that the scores were negatively correlated with risk factors and positively correlated with protective factors in the training set (**Figure 3F**) and verification set (**Figure 4F**).

**Figure 5:**
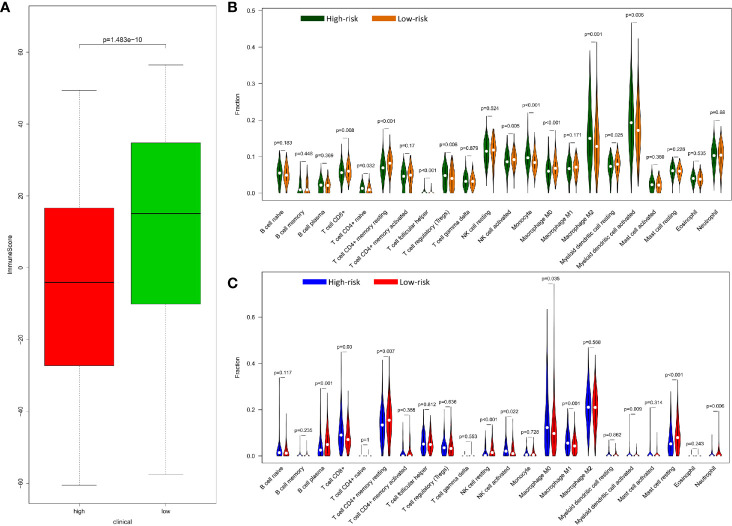
Relationship of between immune and prognostic risk in CRC. **(A)** Immune microenvironment score of high-risk and low-risk group in GEO data with wilcox.test. **(B)** 22 types of immune cells infiltration of high risk and low risk group in GEO data by R package “e1071”, “parallel”, and “preprocessCore”. **(C)** 22 types of immune cells infiltration of high-risk and low-risk group in TCGA data by R package “e1071”, “parallel”, and “preprocessCore”.

The AUC of the ROC curve measured the specificity and sensitivity of the model. The model of training set (AUC = 0.702) indicated that this model had a good evaluation accuracy (**Figure 3G**). The TCGA validation centralized model AUC = 57 (**Figure 4G**), which did not show the good evaluation of the model. The AUC of ROC curve was 0.754 in GSE12954 set **(** **Figure 4M** **)** and 0.718 in GSE17536 set **(** **Figure 4P** **)** respectively. The two validation sets showed good evaluation accuracy. The ROC curves of the 3-year survival (AUC = 0.683 in **Figure 3H**) and 5-year survival (AUC = 0.677 in **Figure 3I**) in the training set showed good authenticity. However, the authenticity of the 3-year survival (AUC = 0.51 in **Figure 4H**) and 5-year survival (AUC = 0.57 in **Figure 4I**) in the validation set was not high.

Furthermore, we evaluated the relationship between each prognostic risk-related DEAG and overall survival. In the training set, the low expression of BCL2, DAPK2, CASP1, CASP3, HDAC1, PRKAB1, MTMR14, BID, BIRC5, and MYC was more beneficial to the survival of CRC patients, as well as the high expression of ULK3, CAPN2, DAPK1, RAF1, and BAG3 corresponded to longer survival (**Figure 3J**). The low expression of CASP1, HDAC1, BIRC5 was better to the survival of CRC patients in the verification set. However, other DEAGs did not show the meaningful change to survival of CRC (**Figure 4J**). GSEA analysis showed that DEAGs of low-risk in KEGG analysis were enriched autophagy associated pathway but the results were not statistically significant (**Figure S5**).

### Immune Score and Immune Cell Infiltration of Risk Model in Colorectal Cancer

In terms of evaluating tumor immunity, the immune score was higher in the low-risk group than in the high-risk group in the training set (**Figure 5A**) but there was no significant difference in the validation set (**Figure S6**). With regard to the infiltration level of 22 immune cells in the high-risk and low-risk groups of the training set and validation set, the content of CD8 + T cell, active NK cell and macrophages M0 was more in the low-risk group of the training set but in the high-risk group the validation set. Active dendritic cell was more in the high-risk group of two sets. The content of CD4+ T cell naïve, Tregs, T cell follicular helper, Monocyte, and Macrophage M2 was only more in the high-risk group of the training set, and macrophages M1 was only more in the high-risk group of the validation set. On the other hand, the content of resting memory CD4 + T cell was more in the low-risk group of two sets. The content of resting dendritic cell was just more in the low-risk group of the training set, and the content of plasma cell, resting NK cells, resting mast cells, and neutrophil cells was only higher in the low-risk group of the validation set. The remaining immune cells were not significantly different between the high and low risk groups. (**Figures 5B, C**).

### The Expression of Prognosis-Related Differentially Autophagy-Related Gene Verification in Oncomine Database and Human Protein Atlas

**Figure 6:**
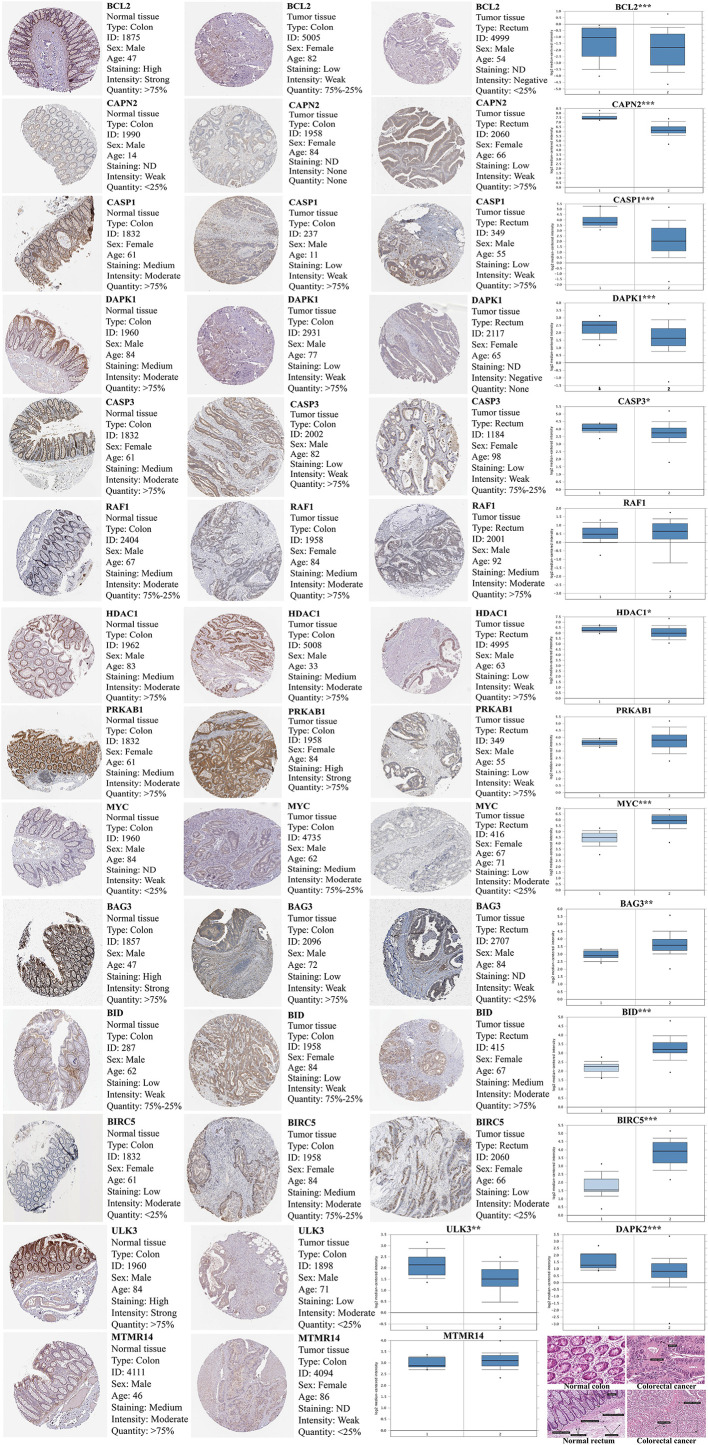
The expression of 15 prognosis-related DEAGs in Oncomine database (https://www.oncomine.org/resource/main.html) and Human Protein Atlas database (https://www.proteinatlas.org/). The blue box plot was a visualization of gene expression from Oncomine database. (“1” represented normal tissue and “2” represented tumor tissue.) The results of immunohistochemistry and HE staining were obtained from the HPA database. *P < 0.05, **P < 0.01, ***P < 0.001, and ****P < 0.0001.

Compared with normal tissues, BCL2, ULK3, DAPK2, CAPN2, CASP1, DAPK1, CASP3, RAF1, HDAC1, PRKAB1, and MTMR14 are lowly expressed in CRC, and BAG3, BID, BIRC5, and MYC are highly expressed. Verifying the expression of key DEAGs in the Oncomine database, we found that the expression levels of most of 15 prognosis-related DEAGs were verified in Oncomine database and the same to our results. However, the expression of RAF1, PRKAB1, and MTMR14 was no obvious difference. Through the results of immunohistochemistry in Human Protein Atlas (HPA) database, we found that these 15 DEAGs were consistent with our results excluding DAPK2 without data in HPA database (**Figure 6**).

### Screening of Differentially Autophagy-Related lncRNA and Reconstruction of Risk Prognosis Models

**Figure 7:**
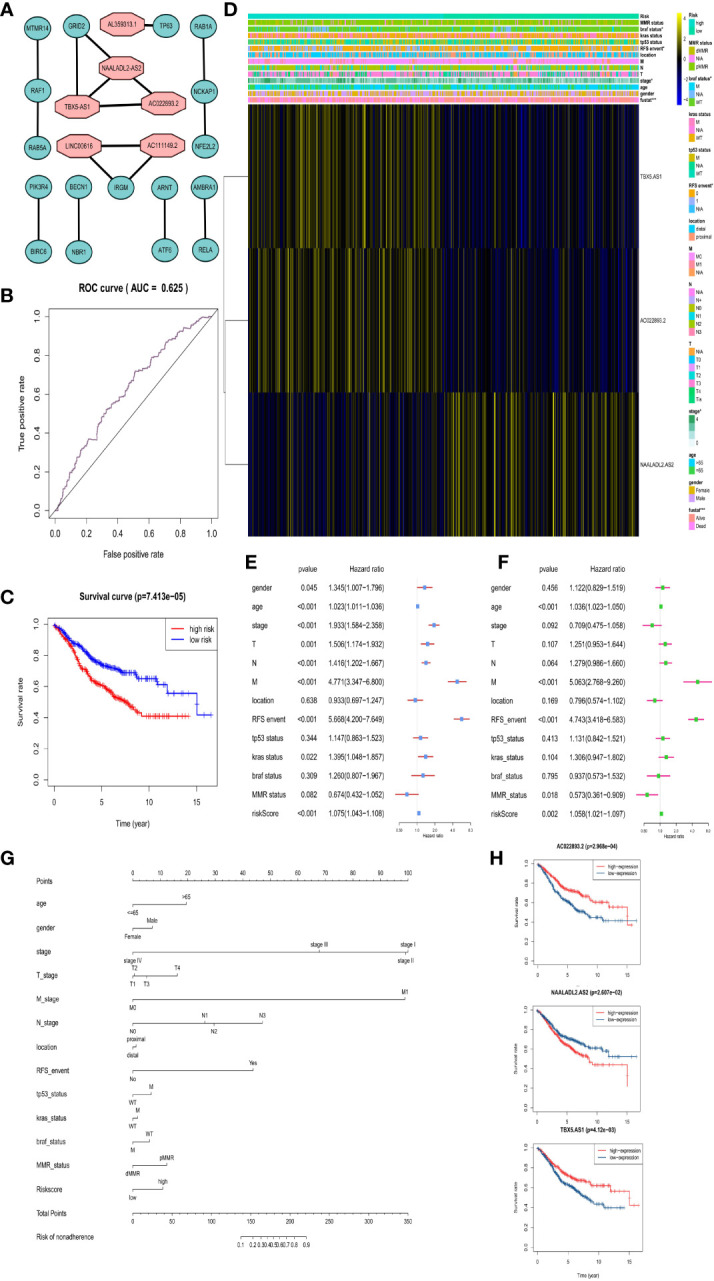
Risk prognosis model construction of three prognostic risk-related DAR-lncRNAs in GEO data. **(A)** Co-expression network diagram of DAR-lncRNAs and DEAGs in the Cytoscape. **(B)** ROC curve of risk prognosis model in the training set by R package “survivalROC”. **(C)** Survival curve comparing high-risk and low-risk groups by R package “survival”. **(D)** Heat map of prognostic DAR-lncRNAs and clinical parameters at high-risk and low-risk groups by R package “pheatmap”. **(E)** The univariate cox forest map of clinical characteristics in the training set by R package “survival” and “forestplot”. **(F)** The multivariate cox forest plot of clinical characteristics in the training set by R package “survival” and “forestplot”. **(G)** The nomogram baseline of multivariate cox analysis by R package “rms”. **(H)** The survival curve of three prognostic risk-related DAR-lncRNAs by R package “survival”. *P < 0.05, **P < 0.01, ***P < 0.001, and ****P < 0.0001.

In addition, we obtained that six DAR-lncRNAs in CRC are AC022893.2, AC111149.2, AL359313.1, LINC00616, NAALADL2-AS2, and TBX5-AS1 by WGCNA (**Figure 7A**).

**Figure 8:**
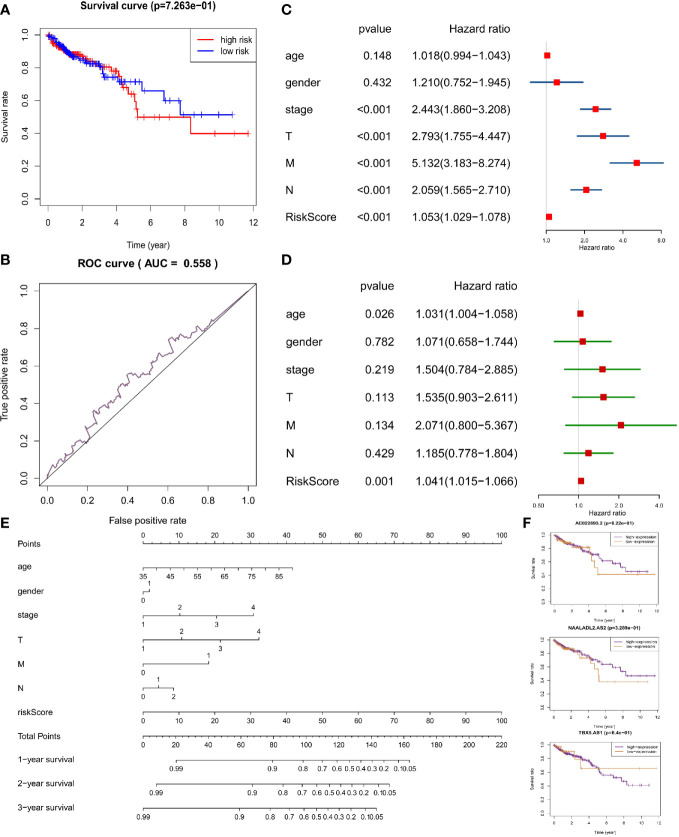
Risk prognosis model verification of three prognostic risk-related DAR-lncRNAs in TCGA data. **(A)** Survival curve comparing high-risk and low-risk groups by R package “survival”. **(B)** ROC curve of risk prognosis model in the verification set by R package “survivalROC”. **(C)** The univariate cox forest map of clinical characteristics in the verification set by R package “survival” and “forestplot”. **(D)** The multivariate cox forest plot of clinical characteristics in the verification set by R package “survival” and “forestplot”. **(E)** The nomogram baseline of multivariate cox analysis by R package “rms”. **(F)** The survival curve of three prognostic risk-related DAR-lncRNAs by R package “survival”.

In the same way, we would get three prognosis-related DAR-lncRNAs (Risk model = NAALADL2.AS2*0.202782 − AC022893.2*0.1573 − TBX5.AS1*0.02432) and also build the risk prognosis model (**Figure S7**) in the training set (AUC = 0.625 in **Figure 7B**) and verified in the verification set (AUC = 0.558 in **Figure 8B**), and the overall survival was also longer in the low-risk group from the training set (HR = 0.562987, 95% CI = 0.424013–0.747511) (**Figure 7C** **)** and validation set (HR = 0.583937, 95% CI = 0.412720–0.826185) (**Figure 8A**). Then we checked the relationship between clinical factors and the model, and found that in the training set, the dead status of patients, stage 3–4, the occurrence of RFS events, and the mutation status of the BRAF gene were all closely related to the high prognostic risk (**Figure 7D**). Through univariate cox regression analysis, RFS event, M stage, pathological stage, risk score, N stage, age, T stage, KRAS gene status, and gender were all risk factors for prognosis of CRC patients (**Figure 7E**). Multivariate regression analysis also reported that RFS event, age, M stage, and risk score were risk factors for CRC prognosis. It was also found that MMR status was a protective factor (**Figure 7F**). In the TCGA verification set, we have not found that clinical factors have a great relationship with risk prognosis models. However, TNM stage and pathological stage are risk factors through univariate cox regression analysis (**Figure 8C**). However, multivariate regression analysis only obtained risk score and age as dangerous factors for CRC prognosis (**Figure 8D**). The nomogram baseline was also used to display multivariate regression analysis results (**Figures 7G** and **8E**). The survival curves of three prognostic risk-related DAR-lncRNAs in the training set and TCGA set were shown in the **Figure 7H** and **Figure 8F**.

Furthermore, the microenvironmental immune score did not show correlation with the risk model in the training set (**Figure S8A**) and the validation set (**Figure S8B**). In the training set (**Figure S8C**) and verification set (**Figure S8D** **)**, there were still no difference in the relationship with 22 types of immune cells.

## Discussion

In the process of CRC treatment, the enhancement of autophagy can make CRC cells survive under the stress state of lack of nutrition and energy, which strengthens the resistance to radiotherapy and chemotherapy. In biological therapy, cell autophagy is continuously amplified and can cause programmed cell death of CRC cells (20). Therefore, autophagy can be used as a predictor in the treatment and prognosis of CRC. We downloaded the transcriptome data from the GEO and TCGA databases to screen out DEAGs and DAR-lncRNAs to construct risk prognosis model respectively. We used no supervised cluster to classify CRC patients and evaluate the relationship between clinical characteristics and immune response of each group. In order to develop a new prognosis and efficacy evaluation tool of CRC, we got 15 key DEAGs related to prognosis and compared results among normal tissues. We find that BCL2, ULK3, DAPK2, CAPN2, CASP1, DAPK1, CASP3, RAF1, HDAC1, PRKAB1, and MTMR14 were lowly expressed in CRC, and BAG3, BID, BIRC5, and MYC were highly expressed. These different results of RAF1, PRKAB1, and MTMR14 between our results and Oncomine database need to further study due to limited sample size.

As a conclusion, evidence of 15 prognostic-related DEAGs are associated with autophagy has been confirmed in some cancers. BCL2 is a marker protein of apoptosis (21). In experiments using conditioned reprogrammed cells from patients with CRC, the expression of BCL2 in CRC was reduced to regulate the formation and apoptosis of autolysosomes. A synergistic growth inhibitory effect was observed in the cells (22). The promoter of autophagy-related gene ULK3 will promote the proliferation of NCoR-enriched glioblastoma cells (23). In the early years, researchers discovered that autophagy defects caused by the role of the tumor suppressor DAPK in the autophagy pathway played a pathogenic role in the formation of cancer (24). DAPK1 gene silencing can prevent the autophagy to induce apoptosis by Dihydroartemisinin in cholangiocarcinoma (25). The low expression of DAPK2 in hepatocellular carcinoma (HCC) attenuates the protective effect of DNA damage mediated by autophagy (26). RAF1 is one of the targeted molecules for sorafenib in the treatment of HCC affecting the proliferation, autophagy, and apoptosis of HCC cells (27). When the expression level and activity of PRKAB1 decrease, it can inhibit the process of autophagy induction of hepatocellular carcinoma (28). Knocking out MTMR14 can induce autophagy to promote liver cancer cell apoptosis and inhibit cell migration ability (29). Similarly, MYC is highly expressed in glioblastoma that can induce autophagy to inhibit apoptosis of hypoxic GBM cells (30). For the research of HDAC1, it has been found that inhibition of HDAC1 can enhance DNA-mediated cell death of CRC cells (31). These conclusions were consistent with our results.

CAPN2 is involved in tumor autophagy (32). But the role of autophagy gene CAPN2 in CRC is not clear. We found that lowly expressed CAPN2 was associated with the CRC poor prognosis. CASP1 mediated autophagy to regulate mitochondrial dysfunction can also effectively control the immune system disorders of inflammatory immune diseases (33). However, the role of CASP1 in tumor autophagy is still unclear. CASP3 is mainly involved in the process of apoptosis, which is involved in the autophagy and apoptosis of various tumors (34). We found that low expression of CASP1 or CASP3 corresponded to longer survival in CRC. Highly expressed BAG3 and BID were closely related to good prognosis of CRC. Other researchers found different conclusions in other tumors. Down-regulation of BAG3 by autophagy inhibitors can enhance mitochondrial-dependent apoptosis activity of Jurkat T cells (35). BID is a pro-apoptotic factor that also plays an important role in autophagy (36). BID is increased in HCC to induce enhanced autophagy, thereby accelerating cell apoptosis (37). Zhang et al. found that down-regulation of BIRC5 can induce double cell death of head and neck cell carcinoma apoptosis and autophagic cell death (38). But Lin et al. found that BIRC5 can negatively regulate autophagy to maintain the DNA integrity of tumor cells (39). These genes need further study in CRC.

The three prognostic DAR-lncRNAs we obtained are AC022893.2, NAALADL2.AS2, TBX5.AS1. AC022893.2 and NAALADL2.AS2 have not found evidence related to tumor prognosis in the currently published studies. We found for the first time in CRC that they co-expressed with DEAGs and may be involved in the process of autophagy in CRC. TBX5.AS1 is significantly down-regulated in damaged heart tissue of tetralogy of Fallot (40). TBX5.AS1 has been found to be a prognostic factor for lung adenocarcinoma (41). We found that TBX5.AS1 is involved in autophagy. These three lncRNAs are risk factors for CRC prognosis and worth further study.

It is an established fact that the low-risk group survives longer. Low TNM and pathological stages predict better survival and have been widely used in the diagnosis and treatment of CRC. We also found that low-stage, no lymph nodes and distant metastasis predict a better prognosis. Advanced age (>65) and men may have a worse prognosis. Current studies have indicated that dMMR is an independent protective factor in stage II CRC, while it is a risk factor in advanced CRC (42). In our data, dMMR is a protective factor in the overall comparison of this study. The possible reason is that the proportion of advanced CRC is low. The TCGA dataset is not as sensitive to model validation as the training set data. This may be due to individual differences between patients, different sample acquisition, detection and analysis method, the limited sample size. The analysis of each prognosis-related DEAG and survival found that a single prognosis-related DEAG can also predict the prognosis, as discussed in the previous paragraph.

In recent years, the status of BRAF, KRAS, and MMR, and the occurrence of RFS events can also be used as tools for evaluating efficacy and prognosis of CRC (2). KRAS is generally recognized tumor suppressor gene, and its mutations usually lead to the poor prognosis and low response of chemotherapy in CRC (43). BRAF is one of the key genes of the MAPK/ERK signaling pathway, which is an important pathway for regulating tumor proliferation and apoptosis. Its mutation will lead to the abnormal activation of the MAPK/ERK signaling pathway, thereby promoting tumor proliferation and inhibiting apoptosis (44). Our results indicated that the mutational status of these genes also put patients at greater risk of poor prognosis. dMMR/MSI-H status is one of the predictive indicators of CRC immunotherapy and chemotherapy. dMMR/MSI-H CRC patients undergoing XELOX or FOLFOX chemotherapy have a higher ORR (45). When the genes expression of MMR is lost or weakened, it will cause DNA mismatch aggregation, and the microsatellite repeat sequence in the cell will change, resulting in genetic instability (46). We found that dMMR is associated with good prognosis, although it is not statistically significant. Therefore, these factors may be the independent predicted tool of prognosis in CRC.

Tumor immunity always exists in the prognosis, development, and therapeutic response of CRC (47). The higher immune microenvironment score of the tumor prognosis, which is consistent with our findings. The subtypes of tumor-associated macrophages (TAM) in the tumor microenvironment is associated with the good or poor prognosis of several tumor types. Lowly infiltrating macrophage M2, highly infiltrating macrophage M0 and macrophage M1 cause lower risk of prognosis and better therapeutic response of treatment in CRC (48). Plasma cells, resting memory CD4+ T cells and resting NK cells are good prognostic factors for various tumors (49). This is consistent with the CRC results. These results showed that the model we constructed and the prognostic-related DEAGs not only can predict the changes in the level of these immune cells but also prognosticate the efficacy and prognosis of CRC immunotherapy.

Dendritic cells are the most important antigen-presenting cells in immunity. The central link connecting the initial and secondary immunity can be used for tumor immunotherapy. Dendritic cell vaccines have been tested in clinical trials of various tumors (50). NK cells are known to kill tumor cells (51). However, active dendritic cells and active NK cells increased in the CRC prognosis high-risk group, which is contrary to most studies. Resting mast cells and neutrophil cells have been shown to be poor prognostic factors in existing CRC studies (52, 53). This is also inconsistent with our results. These opposite results may be due to the limited sample size. Also, the individual differences between patients, pathological stages, and molecular subtypes will all cause such opposite results. In other words, the higher abundance of immune cell infiltration may predict the better response of immunotherapy. The level of infiltration of these immune cells is closely related to the risk model based on DEAGs, so these DEAGs are also potential markers for predicting the efficacy of immunotherapy.

In the final analysis, we use DEAGs to build a risk prognosis model for CRC, and then use DAR-lncRNAs to build the model again. The influence trend of the two models on CRC prognosis is consistent. This provided the evidence for the clinical application of these autophagy-related molecules as a tool for evaluating the therapeutic efficacy and prognosis of CRC, and they can also be used as biomarkers for prognosis and treatment of CRC. This provided evidence that shows autophagy-related molecules can be used as a tool to evaluate the therapeutic efficacy and prognosis of CRC. Also, it is reliable to use autophagy related molecules as biomarkers for prognosis and treatment of CRC.

## Data Availability Statement

Publicly available datasets were analyzed in this study. These data can be found here: The Cancer Genome Atlas (https://portal.gdc.cancer.gov/); the NCBI Gene Expression Omnibus (GSE40967).

## Author Contributions

YY and MF contributed to finish all the manuscript. LB, MZ, and KZ contributed to the data collection and collation. WLi, WLe, and NZ analyzed the statistics of the data. JH and QL made the figures. All authors contributed to the article and approved the submitted version.

## Conflict of Interest

The authors declare that the research was conducted in the absence of any commercial or financial relationships that could be construed as a potential conflict of interest.
