# Supplementary material for: The Effects of Autophagy-Related Genes and lncRNAs in Therapy and Prognosis of Colorectal Cancer
Source: Front Oncol. 2021 Mar 11;11:582040. doi: 10.3389/fonc.2021.582040 (PMC7991845; doi:10.3389/fonc.2021.582040)
Supplement: Supplementary file 1 [file DataSheet_1.docx]

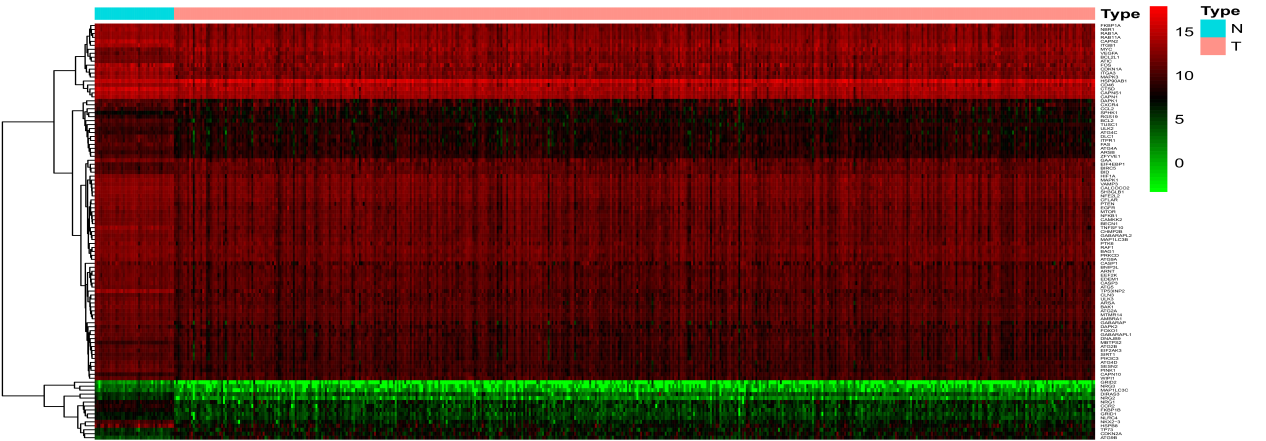


**Figure S1.** DEAGs between tumor group and normal group by heat map. (p＜0.05)


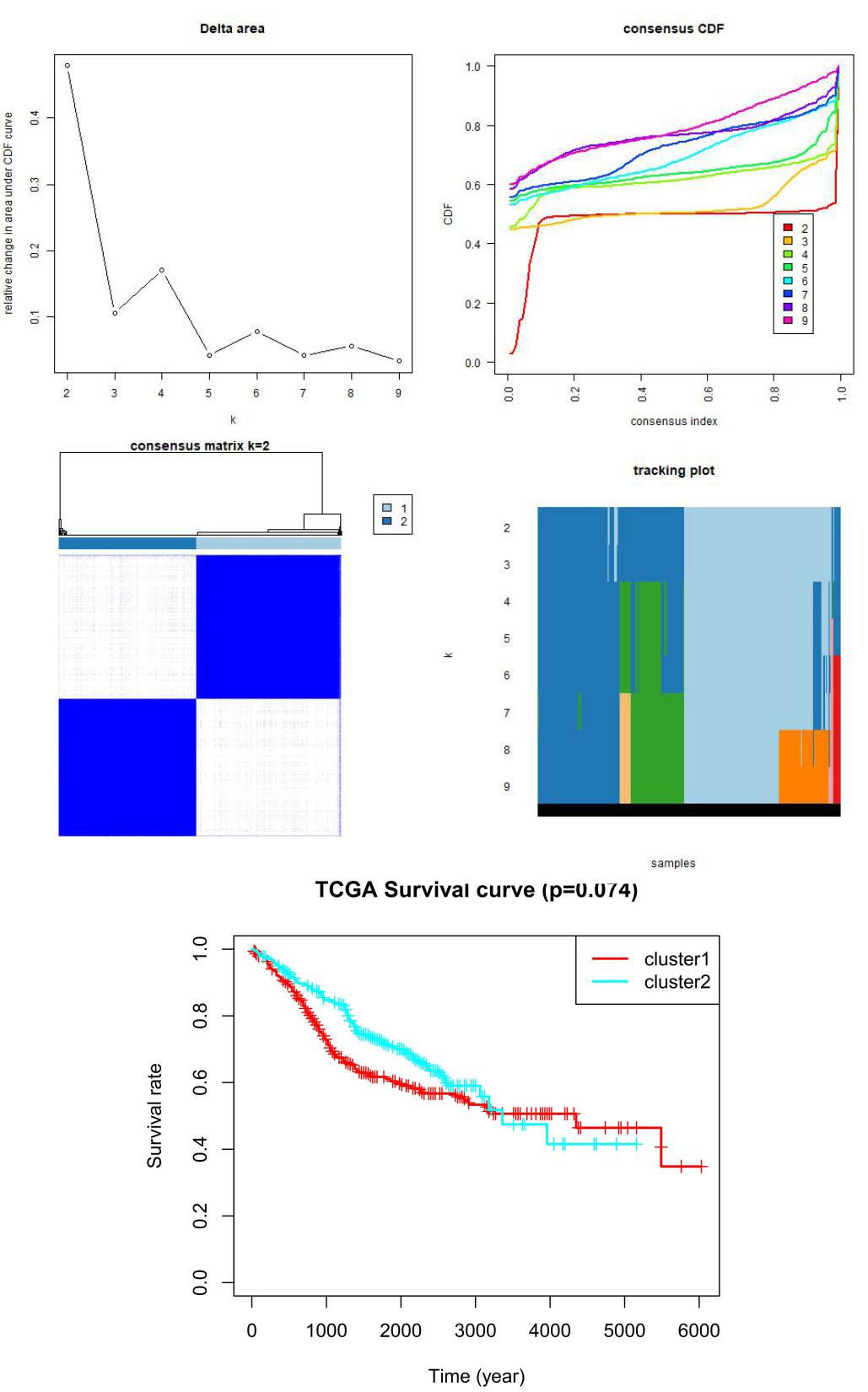


**Figure S2.** Unsupervised cluster analysis of CRC in TCGA set and survival time in each cluster.


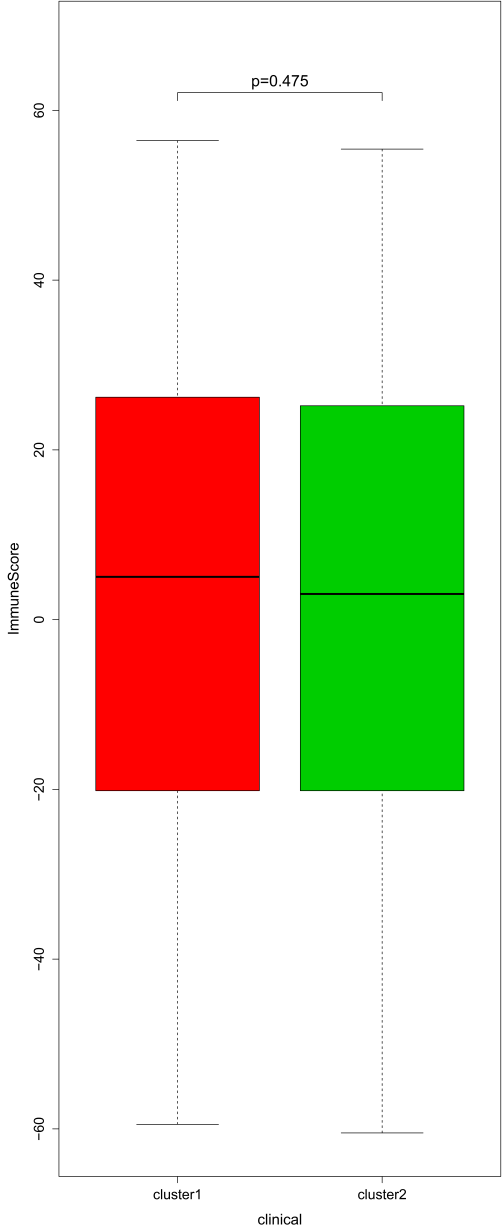


**Figure S3.** Immune score between the two clusters in the GEO data.


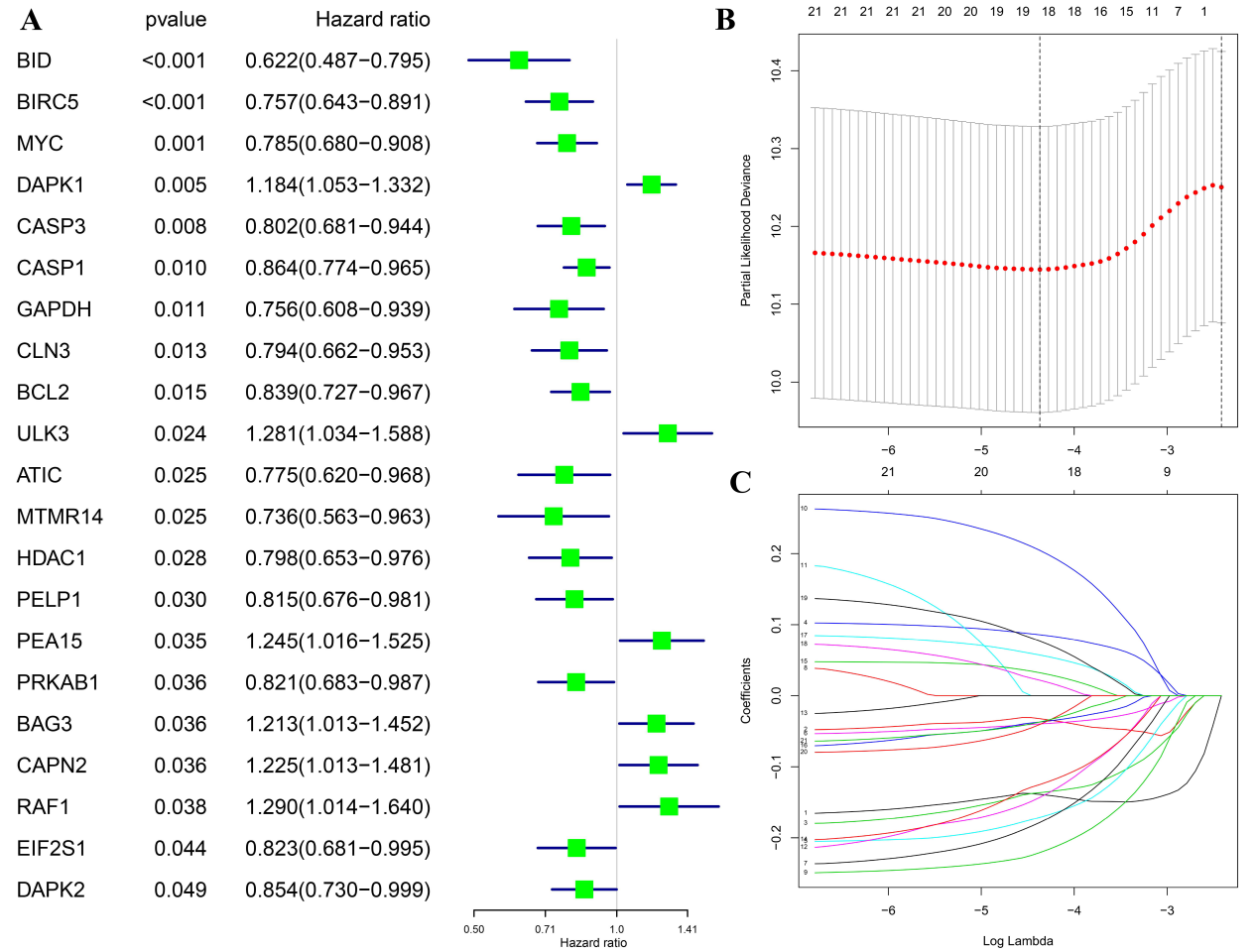


**Figure S4.** Risk prognosis model based on DEAGs. **A.** The forest plot of 15 prognostic risk-related DEAGs. **B.** The civit chart of Lasso regression. **C.** The lambda chart of Lasso regression.


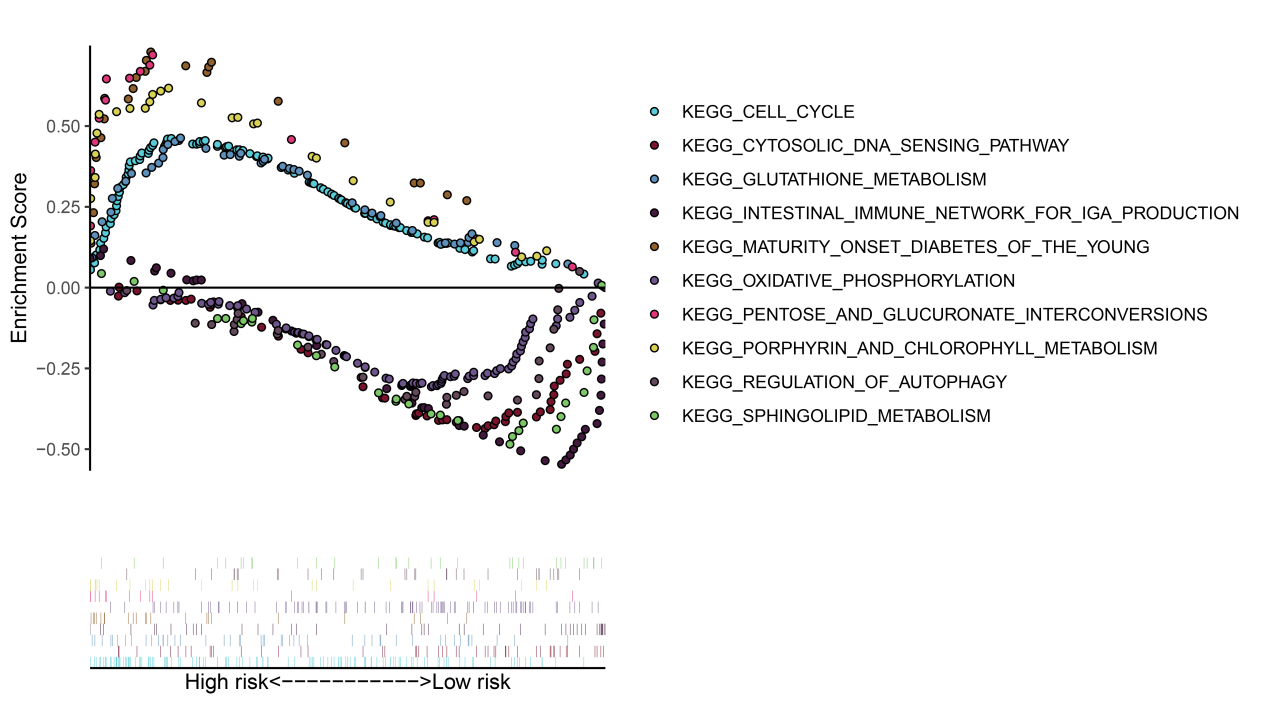


**Figure S5.** KEGG pathway analysis about risk prognosis model by GSEA.


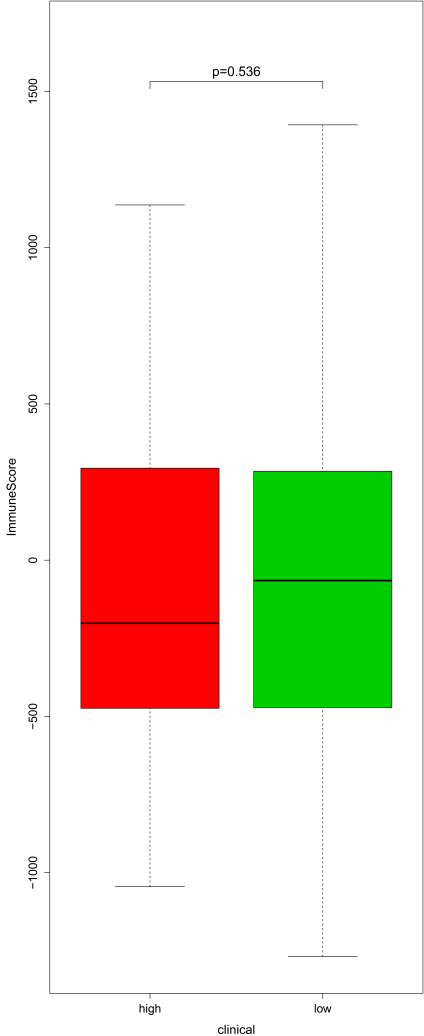


**Figure S6.** Immune score between high-risk and low-risk group based on DEAGs in the GEO data.


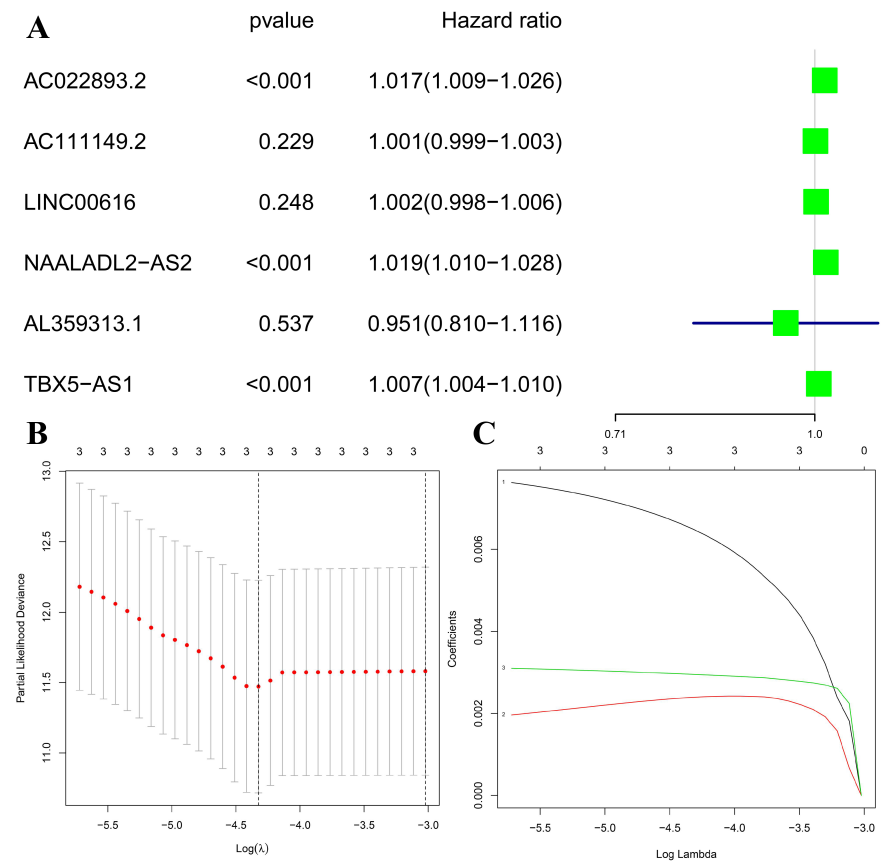


**Figure S7.** Risk prognosis model based on DAR-lncRNAs. **A.** The forest plot of 3 prognosis-related DAR-lncRNAs. **B.** The civit chart of Lasso regression. **C.** The lambda chart of Lasso regression.

**
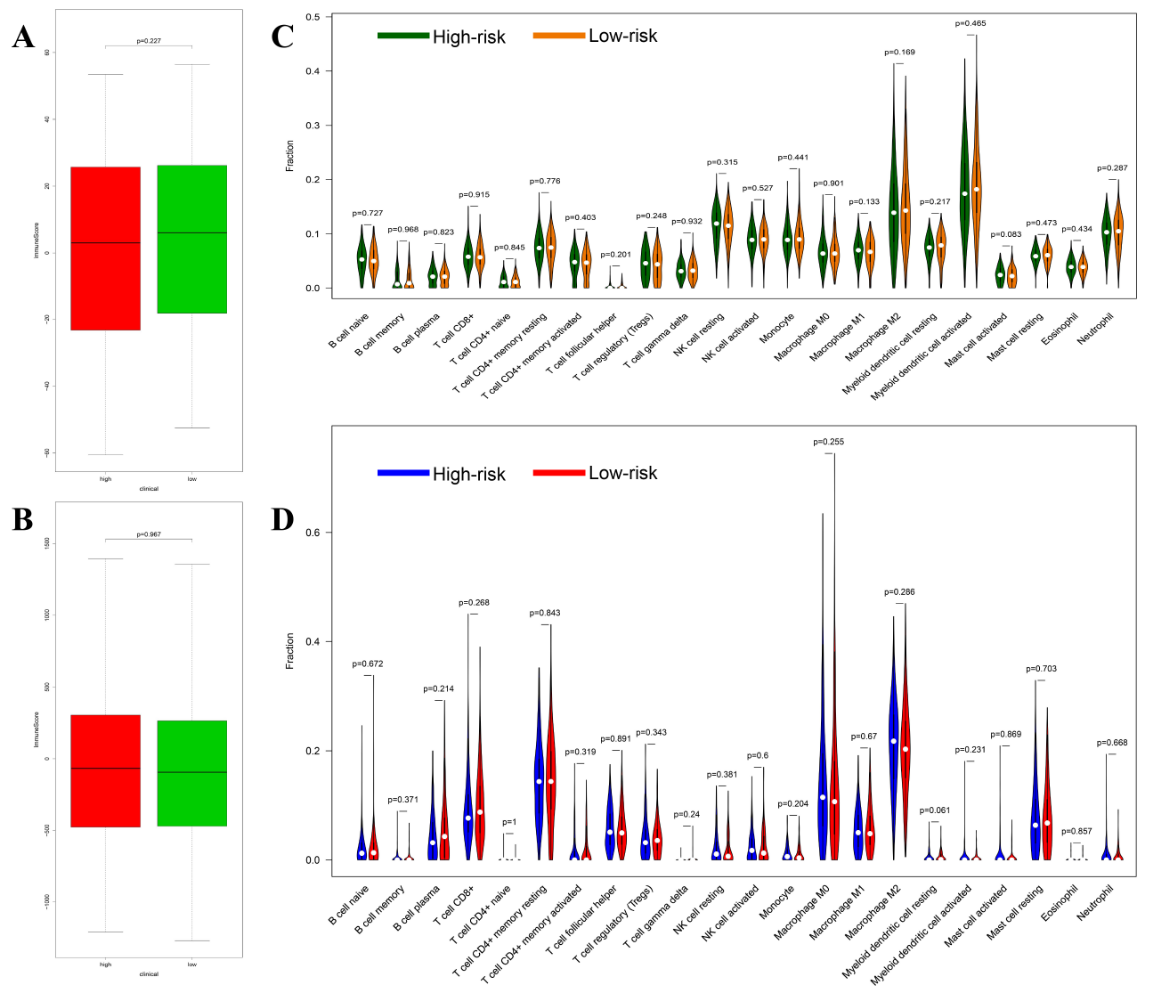
**

**Figure S8.** The correlation between immune and risk score based on DAR-lncRNAs. **A.** Immune score between high-risk and low-risk group based on DAR-lncRNAs in the GEO data. **B.** Immune score between high-risk and low-risk group based on DAR-lncRNAs in the TCGA data. **C.** 22 types of immune cells between high-risk and low-risk group based on DAR-lncRNAs in the GEO data. **D.** 22 types of immune cells between high-risk and low-risk group based on DAR-lncRNAs in the TCGA data.
